# Regional variations of contraceptive use in Bangladesh: A disaggregate analysis by place of residence

**DOI:** 10.1371/journal.pone.0230143

**Published:** 2020-03-25

**Authors:** Md. Kamrul Islam, Md. Rabiul Haque, Prianka Sultana Hema

**Affiliations:** 1 Prentice Institute for Global Population and Economy, University of Lethbridge, Alberta, Canada; 2 Department of Population Sciences, University of Dhaka, Dhaka, Bangladesh; Shahjalal University of Science and Technology, BANGLADESH

## Abstract

This study advances current knowledge on contraceptive use in Bangladesh by providing new insights into the extent of regional variations in contraceptive use across rural and urban areas of Bangladesh. We examined the regional variations in contraceptive use among 15,699 currently married women ages 15–49 years using data from the 2014 Bangladesh Demographic and Health Survey (BDHS). Multivariate logistic regression models of contraceptive use were calibrated with sociodemographic attributes and cultural factors. Based on the aggregate sample (i.e., rural and urban combined), we found significant regional variations in contraceptive use across the administrative divisions in Bangladesh. Based on a disaggregate sample (i.e., rural and urban separately), we found that there were significant differences in divisional variations in contraceptive use in rural areas. In contrast, no significant variation in contraceptive use across divisions in urban areas of Bangladesh was found. More specifically, among women living in rural areas, the Rajshahi and Rangpur divisions had higher odds of contraceptive use than the Barisal division, whereas the Chittagong and Sylhet divisions had much lower odds of contraceptive use even after adjusting for selected sociodemographic attributes and cultural factors. A separate analysis of the divisional variations in usage of modern methods of contraception also revealed similar findings with only one exception. Findings of this study provide an evidence-based direction for adapting a pragmatic approach to reducing the divisional disparity of contraceptive use in rural areas of Bangladesh.

## Introduction

Family planning programs in Bangladesh have generated much interest among researchers and policy makers globally because of their outstanding success in increasing the contraceptive prevalence rate (CPR)—even in the context of a Muslim-majority country characterized by higher poverty, a lower literacy rate, and a lower level of women’s autonomy [1–4]. The CPR in Bangladesh has increased to 62.4% in 2014 from a mere 8.0% in 1975 [4–5]. The remarkable increase in the CPR has not only contributed to the decline of the total fertility rate (TFR) to 2.3 children per woman in 2014, from 6.3 children per woman in 1975 [4], but it has also facilitated large declines in maternal mortality (from 574 per 100,000 live births in 1990–91 to 170 in 2013) and infant mortality (from 88.0 per 1000 live births in 1993–94 to 38.0 in 2014) in Bangladesh [6,7]. However, over the last few years, the increasing trend in CPR in Bangladesh has become stalled (e.g., 61.0% in 2011, 62.4% in 2014 and 62.0% in 2017) and, consequently, the declining trend in TFR has also become stagnant at 2.3 children per woman from 2011 to 2017 [5,8].

In order to slow population growth and further improvement in maternal and child health, the Government of Bangladesh has a target to increase CPR 75% by 2021, thus achieving the below replacement level of fertility (i.e., less than 2.1 children per woman). However, within the current context the latter has become a daunting challenge [4,9]. To maintain the increasing trend of the CPR by addressing barriers of contraceptive usage, the Government of Bangladesh needs to adapt an evidence-based pragmatic approach in its family planning programs. This study aims to contribute to this cause by identifying the regions (in this case administrative divisions) where greater efforts are needed to increase the CPR and by distinguishing the factors that have a strong influence in CPR in rural and urban areas of Bangladesh. It should be mentioned that there were seven administrative divisions (a first-order administrative unit comprised of several districts, as defined by the Government) in Bangladesh prior to 2015—Barisal, Chittagong, Dhaka, Khulna, Rajshahi, Rangpur and Sylhet. On September 2015, Mymensingh became the 8th administrative division in Bangladesh. Disaggregated data on this division about different factors of contraceptive use are not available in the 2014 BDHS report.

The success story of family planning programs in Bangladesh led many researchers to examine the determinants of contraceptive use in Bangladesh [4, 10–15]. In general, the findings of these studies showed that the women’s age [16], education [16, 17], employment status [11, 18], wealth index [11], living in urban areas [11], husband’s education [18], desire for smaller family size [16], discussion about family planning with husband [17], visit of family planning workers within the last six months [18, 19], and media exposure [11, 16] were positively associated with contraception use in Bangladesh. In addition, significant variations in contraception usage were found across religion [18], division [16], number of children that had died [17], and number of children ever-born [11, 17]. Another strand of literature has looked at rural—urban differentials of contraceptive use in Bangladesh [4, 13, 17, 20]. These studies consistently reported that urban areas had a higher prevalence of contraceptive use than rural areas. Age, religion, permission to go to a hospital/health center, increasing access to family planning messages, and women’s participation in the family decision-making process influenced the contraceptive use for women living in rural areas of Bangladesh to a large extent [4, 15, 17].

Concerning the mechanisms through which the above mentioned predictors influence contraception use, studies have shown that *adolescent women* have higher use of contraception than older women because of their greater awareness about both preventing unwanted pregnancy and their desire to postpone childbearing [21, 22]. Women ages 35 years and above have a lower rate of contraception use because of their greater concern about self-perceived health complications [23–25]. Women with a higher level of *education* are more likely to use contraception than women with lower level of education due to their greater awareness about the negative consequences of having more children on both maternal and child health [18, 26]. *Employed women* are also more likely to use contraception than unemployed women to avoid the negative consequences of having more children on their career and future aspirations [27]. Women belonging to a higher *wealth index* are more like to have reduced risk of the death of their children because of their better access to maternal and child healthcare. This, in turn, motivate them to have fewer children [28, 29]. In addition, women with a higher socioeconomic status are in a better position to play a greater role in the family decision-making process. Hence, they are more able to translate their desire of having fewer children into the reality by use of contraception [11, 16, 17].

Among other predictors that influence contraceptive use, women’s *attitude towards a smaller family size* motivates them to use contraception in order to prevent unwanted pregnancy [16]. Women who have experienced the *death of their child* are less likely to use contraception due to a perceived higher risk of death of their children even after giving birth [17]. Greater *access to media* (radio/TV/newspaper) plays an important role in increasing contraceptive use among couples, as it creates awareness about the negative consequences of having more children on both maternal and child health [11, 16]. From the family planning program perspective, the *visit of family planning workers* to women living in rural areas makes contraceptive materials more accessible to women and facilitates addressing the side effects of contraceptive use, particularly in hard-to-reach areas, which eventually translate into a higher contraception use [18, 19]. *Religious teachings* related to family, sexual relations, and family planning influence contraceptives use among its followers [30–32]. For instance, historically Catholics had a lower rate of contraceptive use than Protestants due to prohibitions of contraceptive use by the Roman Catholic Church [33]. Muslims also had lower contraceptive use compared to the followers of other religions because of their pronatalist ideologies [4]. Women with a higher level of autonomy have a higher rate of contraceptive use because of their greater capability to make decisions and because of their greater awareness about the negative consequences of having more children [14, 17, 34]. Finally, the rate of contraceptive use among couples varies across their *place of residence (i*.*e*., *rural or urban)* because of differences in socioeconomic conditions, the visitation of family planning workers, the level of women’s autonomy and other community-level characteristics [4, 35, 36].

While many studies have looked at the determinants of contraceptive use, to the best of our knowledge, none of the earlier research has explicitly examined the divisional variations in contraceptive use across rural and urban areas of Bangladesh. Based on earlier studies, we know that there are divisional disparities in contraceptive use in Bangladesh [11, 18, 20, 37, 38], but we do not know whether the observed divisional differences in contraceptive use hold true separately for both rural and urban areas. In this context, the objective of this study was to examine the divisional variations of contraceptive use across rural and urban areas of Bangladesh. We expect to find significant differences in contraceptive use across the seven divisions in Bangladesh both at the aggregate level (i.e., rural and urban combined) and the disaggregate level (separately for rural and urban areas). These assumptions are based on the premise that the considerable disparities in socioeconomic and cultural attributes across divisions in rural and urban areas Bangladesh—shown in Table 1—would exert strong influence in determining the regional variations in contraception use.

**Table 1:** Weighted percentage distribution of women aged 15–49 by sociodemographic characteristics in Bangladesh in 2014.

Variable	Barisal (%)	Chittagong (%)	Dhaka (%)	Khulna (%)	Rajshahi (%)	Rangpur (%)	Sylhet (%)
Post-secondary education	12.2	7.3	9.8	8.6	8.3	8.9	5.1
Employed	26.4	26.0	34.4	33.1	42.5	41.5	16.7
Post-secondary education (Husband’s)	15.5	14.8	15.3	13.4	14.3	13.1	7.7
Have access to media	48.7	68.5	69.2	62.3	63.5	52.0	48.7
Visited by family planning workers in last six months	20.5	12.4	19.9	21.0	22.4	21.9	18.4
Child mortality (at least one)	18.3	17.5	15.5	14.7	18.0	16.5	24.7
Ideal number of children (less than three)	76.8	68.8	80.1	87.6	85.5	85.3	61.0
*Total (N)*	*976*	*2886*	*5474*	*1637*	*1894*	*1827*	*1005*

Data Source: The 2014 Bangladesh Demographic and Health Survey

## Materials and methods

This study was based on data from the 2014 Bangladesh Demographic and Health Survey (BDHS), which contains a wide range of information on respondents’ sociodemographic characteristics, marriage and sexual activity, levels of fertility, fertility preferences, fertility regulations (i.e., intentional methods of avoiding pregnancy) [39], child mortality, women’s autonomy and health-seeking behavior. The 2014 BDHS is a nationally representative cross-sectional survey in which data were collected following a two-stage stratified sampling procedure. The total sample size in the 2014 BDHS was 17,863 ever married women ages 15–49 years. However, for this study only currently married women ages 15–49 were selected for analysis because considering the nature of the outcome variable of interest (i.e., contraceptive use), we excluded women who were either pregnant or divorced/widowed/separated at the time of the survey. We also excluded women who mentioned that they had never sex. The percentages of missing cases were very low (less than 0.2%). Hence, *listwise delete procedure* was followed for dealing with missing cases in selecting the final sample size. Thus, the final sample size for this study was reduced to 15,699 currently married women ages 15–49 years.

### Dependent variables

The outcome variable of interest in this study was whether respondents or their husbands were using any contraceptive methods at the time of the survey. The 2014 BDHS collected information on this variable and grouped them into three categories: (1) using modern methods of contraception (e.g., pill, injectable, condom, female sterilization, male sterilization, IUD, implants, vaginal methods); (2) using traditional methods of contraception (e.g., periodic abstinence, withdrawal, other traditional methods); and (3) not using any method of contraception. In reporting the contraceptive prevalence rate, the common practice is to combine both modern and traditional methods of contraception together into one group [40]. Policy makers and program planners widely use this indicator to assess the effectiveness of family planning programs [41]. For this reason, the variable on contraceptive use was coded into two categories: yes (indicating the use of either modern and/or traditional methods of contraception) and no. However, modern and traditional methods of contraception differ widely in terms of effectiveness, and computing unmet needs for family planning is usually done based on the use of modern methods. Hence, we have also treated the *use of modern methods of contraception* (yes/no) as another outcome variable in the multivariate regression analysis to provide a comprehensive understanding on divisional variations of contraception usage in rural and urban areas of Bangladesh.

### Independent variables

The main independent variable of interest in this study was administrative division. As mentioned earlier, there were seven divisions in Bangladesh at the time of the survey in 2014: Barisal, Chittagong, Dhaka, Khulna, Rajshahi, Rangpur, and Sylhet.

### Covariates

A number of sociodemographic and cultural variables were included in the multivariate analysis. *Respondents’ age* at the time of survey was coded into two categories: (1) less than 20 years, and (2) 20 years old and above. Age was coded into two categories to provide better insight for policy makers and program planners about the extent of contraceptive use among adolescents in Bangladesh. This is particularly important in the context of Bangladesh where the prevalence of adolescent motherhood—having children before age 20—is very high compared to other developing countries. For instance, Bangladesh had the highest rate of adolescent fertility in 2017–84 births per 1000 women—compared to other countries in south Asia such as India (25 births), Sri Lanka (15 births), Nepal (62 births), Bhutan (22 births), Pakistan (38 births) and Afghanistan (69 births) [42]. The higher rate of adolescent motherhood has wider consequences on women’s health and careers [3, 43, 44]. Among other variables included in the analysis were *religion* (Islam and others), *education* (no education, primary, secondary, and post-secondary), *employment status* (yes and no), *wealth index* (poorest, poorer, middle, richer, and richest), *husband’s education* (no education, primary, secondary, and post-secondary), *access to media* (yes and no), *family planning workers’ visit in the last six months* (yes and no), *attitude towards the ideal number of children to have* (0–2, 3+, and others: non-numeric), and *women’s autonomy* (high and low). The variable on *access to media* was derived using information on whether respondents’ read newspaper or magazine, listened to the radio, or watched television. Concerning the attitude towards the ideal number of children to have, we included 0–2 children in one category in order to provide better insight into the extent to which women consider having less than three children as the ideal number of children to have. Having 0–2 children on average is considered an important marker of slowing the population growth.

The variable on *women’s autonomy* was generated using information based on three variables: (1) the person who usually decides on the respondent’s health care, (2) the person who usually decides on large household purchases, and (3) the person who usually decides on visits to family or relatives. These three variables on decision making contain information that is grouped into six categories: (1) respondent alone, (2) respondent and husband/partner, (3) respondent and other person, (4) husband/partner alone, (5) someone else, and (6) other. These six categories were used to create a six-point scale, ranging from 1–6, for women’s role in decision making where the first category of *respondents alone* was given the highest score (score of 6) followed by respondent and husband/partner (score of 5), respondent and other person (score of 4), husband/partner alone (score of 3), someone else (score of 2), and other (score of 1). After assigning these scores, a composite index of decision making was generated by combining all three variables together. Thus, the composite index for women’s role in decision making ranges from 3–18, where the higher value indicates a greater role of the respondents in the decision-making process. The Cronbach’s alpha—a coefficient of reliability—for these three variables was 0.78, suggesting very good internal consistency of using it as an index variable. Finally, using the average value of the composite index (12.86) as the cutoff point, a dichotomous variable on women’s autonomy (high and low) was created.

### Analytical approach

A combination of bivariate and multivariate analyses was carried out to examine the divisional variations of contraceptive use in Bangladesh both at aggregate (total sample) and disaggregate levels (i.e., rural and urban separately). In bivariate analyses, the association between contraceptive use and the selected background characteristics of the respondents were examined by reporting test of significance using the Chi-square test. The outcome variable of interest in this study was dichotomous: whether use any contraceptive or not. Hence, multivariate logistic regression models of contraception use were calibrated with sociodemographic attributes and cultural factors. To develop the multivariate logistic regression models, we followed a two-stage procedure for selecting the covariates. First, we conducted a thorough review of literature to identify variables that were found to influence contraceptive use particularly in the context of developing countries. Second, we carried out a bivariate analysis between contraceptive use and the variables selected based on the review of literature. The variables that were statistically significant in the bivariate analysis were included in the multivariate logistic regression models as control variables. Thus, the control variables in all three models were the respondents’ age, religion, education, employment status, wealth index, access to media, family planning workers’ visit within the last six months, attitude towards the ideal number of children to have, women’s autonomy, and the husband’s education. The logistic regression estimates for the total sample were also adjusted for place of residence (i.e., rural or urban) in addition to the above controls.

In the regression models, the Barisal division was used as the reference category because Barisal had the median prevalence of contraceptive use among the seven administrative division of Bangladesh. Using Barisal as the reference would facilitate identifying which divisions had higher rates of contraception usage and which divisions had the lower even after controlling for the sociodemographic attributes and cultural factors. This information will in turn help policy makers and program planners in selecting appropriate divisions for further program interventions. Logistics regression estimates were reported in the form of odds ratios (OR). Normalized sampling weights were used in the analysis. All analyses were conducted using Stata 16.0.

## Results

Table 2—sample characteristics of the respondents—showed that the overall percentage of contraceptive use (including both male and female contraceptive methods) was 66.8%. Women living in urban areas had higher percentage of contraceptive use than those living in rural areas (69.9% and 65.5% respectively). More than one-third of the women were from the Dhaka division. Among the total respondents, 10.4% were less than 20 years old. Overall, 90.1% women were the followers of Islam, and about one-fourth of the total respondents had no formal education. About one-third women were employed with slightly higher percentage in rural areas. Only 19.1% women mentioned that they had family planning workers’ visit in the last six months; and the percentage was slightly higher in rural areas than urban areas. About one-fifth of women mentioned having three or more children as the ideal number of children to have, and the percentage was higher in rural areas than urban areas. Overall, 63.3% of women had high level of women’s autonomy, with urban areas having a higher percentage of high autonomy than rural areas (Table 2).

**Table 2:** Weighted distribution of women aged 15–49 by background characteristics in Bangladesh, 2014 BDHS^¥¥^.

Variable	Total n (%)	Urban n (%)	Rural n (%)
*Contraceptive use*			
Yes	10479 (66.8)	3088 (69.9)	7392 (65.5)
No	5220 (33.2)	1328 (30.1)	3892 (34.5)
Division			
Barisal	976 (6.2)	278 (6.3)	699 (6.2)
Chittagong	2886 (18.4)	896 (20.3)	1990 (17.6)
Dhaka	5474 (34.9)	1990 (45.1)	3483 (30.9)
Khulna	1637 (10.4)	395 (9.0)	1241 (11.0)
Rajshahi	1894 (12.1)	398 (9.0)	1497 (13.3)
Rangpur	1827 (11.6)	275 (6.2)	1552 (13.8)
Sylhet	1005 (6.4)	183 (4.2)	822 (7.3)
*Age (years)*			
Less than 20	1631 (10.4)	422 (9.6)	1209 (10.7)
20 and above	14068 (89.6)	3993 (90.4)	10075 (89.3)
*Religion*			
Islam	14139 (90.1)	3877 (87.8)	10262 (90.9)
Other	1560 (9.9)	538 (12.2)	1022 (9.1)
*Education*			
No education	3793 (24.2)	808 (18.3)	2986 (26.5)
Primary	4582 (29.2)	1094 (24.8)	3488 (30.9)
Secondary	5949 (37.9)	1772 (40.1)	4177 (37.0)
Post-secondary	1375 (8.8)	742 (16.8)	633 (5.6)
*Employment status*			
Yes	5164 (32.9)	1334 (30.2)	3830 (33.9)
No	10535 (67.1)	3081 (69.8)	7454 (66.1)
*Wealth index*			
Poorest	2833 (18.0)	287 (6.5)	2546 (22.6)
Poorer	2979 (19.0)	238 (5.4)	2741 (24.3)
Middle	3179 (20.2)	499 (11.3)	2678 (23.7)
Richer	3334 (21.2)	1152 (26.1)	2182 (19.3)
Richest	3376 (21.5)	2240 (50.7)	1136 (10.1)
*Husband’s Education*			
No education	4451 (28.4)	848 (19.2)	3603 (31.9)
Primary	4313 (27.5)	994 (22.5)	3320 (29.4)
Secondary	4713 (30.0)	1464 (33.2)	3249 (28.8)
Post-secondary	2221 (14.1)	1109 (25.1)	1112 (9.9)
*Access to media*			
Yes	9901 (63.1)	3820 (86.5)	6080 (53.9)
No	5798 (36.9)	595 (13.5)	5203 (46.1)
*Family planning workers’ visit in last six months*			
Yes	3001 (19.1)	674 (15.3)	2327 (20.6)
No	12698 (80.9)	3742 (84.7)	8957 (79.4)
*Ideal number of children*			
0–2	12343 (78.6)	3686 (83.5)	8656 (76.7)
3+	3108 (19.8)	680 (15.4)	2428 (21.5)
Other (non-numeric)	248 (1.6)	49 (1.1)	199 (1.8)
*Women’s autonomy*			
High	9933 (63.3)	3076 (69.7)	6856 (60.8)
Low	5766 (36.7)	1339 (30.3)	4427 (39.2)
*Total (N)*	*15699*	*4415*	*11284*

^¥¥^ This table includes all respondents: those who were using contraceptives and those who were not using contraceptives

### Bivariate findings

Table 3 presents bivariate findings—for total, urban, and rural samples—on the association between contraceptive use and the selected background characteristics of the respondents. It was found that, among the all respondents, Rangpur had the highest percentage (73.9%) of contraceptive use, which was followed by Rajshahi (73.0%), Khulna (70.5%), Barisal (67.8%), Dhaka (67.1%), Chittagong (59.3%), and Sylhet (54.3%). These differences in contraceptive use by division were statistically significant. Overall, urban areas had a significantly higher percentage of contraceptive use than rural areas (69.9% and 65.5% respectively). A disaggregate analysis by place of residence (urban—rural) showed that there were variations in contraceptive use across divisions in urban areas. However, these variations in contraceptive use were not statistically significant at the 0.05 level. On the other hand, the divisional variations in contraceptive use were statistically significant in the case of rural areas, with Rangpur having the highest percentage of contraceptive use (74.2%) and Sylhet having the lowest (51.8%) (Table 3).

**Table 3:** Association between contraceptive use and selected background characteristics of the respondents, BDHS 2014 (%) ^¥¥^.

Variable	Currently Using Contraceptive					
	Total		Urban		Rural	
	Yes	No	Yes	No	Yes	No
*Division*						
Barisal	67.8 ^a^	32.2	75.1	24.9	64.9 ^a^	35.1
Chittagong	59.3	40.7	70.0	30.0	54.4	45.6
Dhaka	67.1	32.9	68.2	31.8	66.5	33.5
Khulna	70.5	29.5	72.2	27.8	70.0	30.0
Rajshahi	73.0	27.0	72.9	27.1	73.1	26.9
Rangpur	73.9	26.1	72.4	27.6	74.2	25.8
Sylhet	54.3	45.7	65.6	34.4	51.8	48.2
*Place of residence*						
Urban	69.9 ^a^	30.1				
Rural	65.5	34.5				
*Age (years)*						
Less than 20	62.0 ^a^	38.0	70.9	29.1	58.9 ^a^	41.1
20 and above	67.3	32.7	69.8	30.2	66.3	33.7
*Religion*						
Islam	65.9 ^a^	34.1	68.9 ^a^	31.1	64.8 ^a^	35.2
Other	74.1	25.9	77.0	23.0	72.6	27.4
*Education*						
No education	63.8 ^a^	36.2	64.1 ^a^	35.9	63.8	36.2
Primary	67.3	32.7	69.8	30.2	66.5	33.5
Secondary	67.5	32.5	71.4	28.6	65.7	34.3
Post-secondary	70.0	30.0	72.9	27.1	66.7	33.3
*Employment status*						
Yes	70.6 ^a^	29.4	71.1	28.9	70.4 ^a^	29.6
No	64.9	35.1	69.4	30.6	63.0	37.0
*Wealth index*						
Poorest	68.1 ^b^	31.9	69.7	30.3	67.9 ^a^	32.1
Poorer	67.9	32.1	69.3	30.7	67.8	32.2
Middle	66.6	33.4	70.7	29.3	65.8	34.2
Richer	64.9	35.1	70.0	30.0	62.1	37.9
Richest	66.5	33.5	69.8	29.2	60.1	39.9
*Husband’s Education*						
No education	66.1 ^a^	33.9	68.3	31.7	65.6 ^a^	34.4
Primary	68.3	31.7	71.0	29.0	67.4	32.6
Secondary	64.8	35.2	69.8	30.2	62.5	37.5
Post-secondary	69.4	30.6	70.4	29.6	68.3	31.7
*Access to media*						
Yes	67.8 ^a^	32.2	70.6 ^b^	29.4	66.0	34.0
No	65.0	35.0	65.9	34.1	64.9	35.1
*Family planning workers’ visit in the last six months*						
Yes	78.9 ^a^	21.1	78.6 ^a^	21.4	79.0 ^a^	21.0
No	63.9	36.1	68.4	31.6	62.0	38.0
*Ideal number of children*						
0–2	68.9 ^a^	31.1	71.3 ^a^	28.7	67.8 ^a^	32.2
3+	60.4	39.6	63.3	36.7	59.6	40.4
Other (non-numeric)	41.5	58.5	55.1	44.9	38.2	61.8
*Women’s autonomy*						
High	68.4 ^a^	31.6	70.6	29.4	67.3 ^a^	32.7
Low	64.0	36.0	68.3	31.7	62.7	37.3
Total (N)	10479	5220	3087	1328	7392	3892

^a^ Chi-Square Test, p<0.01;

^b^ Chi-Square Test, p<0.05;

^¥¥^ This table includes all respondents: those who were using contraceptives and those who were not using contraceptives

Overall, significant differences were found in contraceptive use by respondents’ age, religion, education, employment status, wealth index, husband’s education, access to media, family planning workers’ visit, attitude towards the ideal number of children to have, and women’s autonomy. A higher percentage of contraceptive use was found among women who were less than 20 years of age, were followers of Islam, had a higher level of education, were employed, belonged to a lower wealth index, were visited by family planning workers, considered two or less than two children as the ideal number of children to have, and had a higher level of autonomy. The separate analysis by place of residence (i.e., rural or urban) showed that the differences in contraceptive use were statistically significant in urban areas in the cases of religion, education, access to media, family planning workers’ visit in the last six months, and attitude towards ideal number of children to have. Nevertheless, in the case of rural areas the differences in contraceptive use by all the background characteristics were statistically significant except for education and access to media (Table 3).

### Multivariate findings

Table 4 presents three logistic regression models of divisional variations in contraceptive use (modern and traditional methods combined) among women ages 15–49 in Bangladesh after adjusting for the selected covariates. Findings based on the total sample showed that significant variations in contraceptive use existed even after controlling for the selected covariates. For instance, women living in Rajshahi had 25.0% [95% CI: 1.07–1.47] higher odds of contraceptive use compared to those living in Barisal. Similarly, Rangpur had 29.0% [95% CI: 1.08–1.54] higher odds of contraceptive use than Barisal. On the contrary, Chittagong and Sylhet had lower odds of contraceptive use (25.0% and 37.0% respectively) than the reference category. In addition, women living in urban areas were found to have 32.0 percent [95% CI: 1.19–1.47] higher odds of contraceptive use compared to their counterparts living in rural areas. The intercept indicates that a woman from Dhaka, ages 20 years or more, had 2.02 times higher odds of contraceptive use compared to the reference category of Barisal assuming that both of them were living in rural areas, were followers of another religion, had no education, had no access to media, did not receive a family planning workers’ visit in last six months, considered having 0–2 children as the ideal number of children to have, and had a low level of autonomy (Table 4).

**Table 4:** Logistics regression estimates of contraceptive use (modern and traditional methods combined) among women aged 15–49 in Bangladesh, 2014 BDHS.

Variables	Full Sample Odds Ratio [95% CI]	Urban Odds Ratio [95% CI]	Rural Odds Ratio [95% CI]
*Division*			
Chittagong	0.75** [0.64–0.88]	0.93 [0.65–1.33]	0.68** [0.57–0.80]
Dhaka	0.98 [0.83–1.16]	0.83 [0.59–1.17]	1.05 [0.87–1.27]
Khulna	1.12 [0.96–1.32]	0.93 [0.66–1.32]	1.20* [1.01–1.43]
Rajshahi	1.25** [1.07–1.47]	0.99 [0.70–1.40]	1.33** [1.12–1.58]
Rangpur	1.29** [1.08–1.54]	0.98 [0.69–1.40]	1.37** [1.13–1.65]
Sylhet	0.63** [0.53–0.75]	0.79 [0.54–1.14]	0.61** [0.50–0.74]
Barisal	[REF]	[REF]	[REF]
*Place of residence*			
Urban	1.32** [1.19–1.47]		
Rural	[REF]		
*Age (years)*			
Less than 20	0.78** [0.67–0.90]	1.07 [0.82–1.40]	0.69** [0.58–0.84]
20 and above	[REF]	[REF]	[REF]
*Religion*			
Islam	0.72** [0.61–0.83]	0.74* [0.56–0.97]	0.71** [0.59–0.86]
Other	[REF]	[REF]	[REF]
*Education*			
No education	0.80* [0.64–0.99]	0.61** [0.43–0.86]	0.84 [0.64–1.12]
Primary	0.91 [0.75–1.12]	0.76 [0.55–1.05]	0.94 [0.73–1.22]
Secondary	0.98 [0.83–1.17]	0.86 [0.66–1.12]	1.02 [0.81–1.29]
Post-secondary	[REF]	[REF]	[REF]
*Employment status*			
Yes	1.16** [1.05–1.29]	1.10 [0.93–1.32]	1.17* [1.03–1.32]
No	[REF]	[REF]	[REF]
*Wealth index*			
Poorest	1.37** [1.13–1.68]	1.07 [0.74–1.54]	1.55** [1.22–1.97]
Poorer	1.30** [1.09–1.55]	1.00 [0.69–1.46]	1.46** [1.18–1.80]
Middle	1.12 [0.96–1.32]	1.02 [0.77–1.35]	1.24* [1.01–1.50]
Richer	1.03 [0.90–1.18]	1.02 [0.83–1.24]	1.09 [0.91–1.32]
Richest	[REF]	[REF]	[REF]
*Husband’s Education*			
No education	0.97 [0.80–1.17]	1.29 [0.93–1.80]	0.83 [0.65–1.04]
Primary	1.04 [0.87–1.23]	1.25 [0.94–1.66]	0.91 [0.73–1.13]
Secondary	0.86 [0.74–1.01]	1.11 [0.87–1.42]	0.74** [0.60–0.89]
Post-secondary	[REF]	[REF]	[REF]
*Access to media*			
Yes	1.15* [1.03–1.29]	1.16 [0.91–1.48]	1.16* [1.02–1.32]
No	[REF]	[REF]	[REF]
*Family planning workers’ visit in the last six months*			
Yes	2.01** [1.74–2.31]	1.62** [1.27–2.07]	2.14** [1.81–2.53]
No	[REF]	[REF]	[REF]
*Ideal number of children*			
*3+*	0.79** [0.71–0.89]	0.76** [0.62–0.94]	0.81** [0.70–0.92]
*Other (non-numeric)*	0.40** [0.29–0.55]	0.56 [0.30–1.02]	0.36** [0.25–0.52]
0–2	[REF]	[REF]	[REF]
*Women’s autonomy*			
High	1.14** [1.03–1.25]	1.25 [0.95–1.33]	1.15* [1.02–1.29]
Low	[REF]	[REF]	[REF]
*Intercept*	*2*.02** *[1*.*52–2*.*69]*	*2*.86** *[1*.*67–4*.*89]*	*1*.99** *[1*.*40–2*.*83]*
*Total (N)*	*15699*	*4415*	*11284*

**p<0.01,

*p<0.05, [REF] indicates reference category

Data Source: The 2014 Bangladesh Demographic and Health Survey

Concerning the regional variations in contraceptive use in urban areas, no significant difference in contraceptive use was found across divisions in Bangladesh. On the other hand, significant variations in contraceptive use by division were found in the case of rural areas of Bangladesh. For example, Rajshahi and Rangpur had higher odds of contraceptive use (33.0% and 37.0% respectively) compared to Barisal. However, Chittagong and Sylhet had lower odds of contraceptive use (32.0% and 39.0% respectively) than Barisal (Table 4). These contrasting findings in the regional variations (i.e., between urban and rural areas) of contraceptive use between urban and rural areas bear huge implications from the perspective of family planning program interventions. This aspect is illustrated in detail in the subsequent section.

The above findings related to divisional variations in contraceptive use across respondents’ place of residence were based on both traditional and modern methods of contraceptive use. However, modern contraceptive methods are the most effective in preventing pregnancy compared to traditional methods [45, 46]. For this reason, family planning programs tend to encourage the use of modern methods. This raises the question of whether the above findings on divisional variations in contraceptive use are similar in the case of modern methods. Table 5 presents logistic regression estimates of the usage of modern methods of contraception among the same respondents, adjusting for the selected covariates. The divisional variations in the use of modern methods of contraception were identical to that of the combined analysis (both traditional and modern methods) with only one exception in the case of rural areas of Khulna division. Consistent with the expectation, the intercepts in the regression models on modern contraceptives were lower compared to the models in Table 4 that included both modern and traditional methods of contraception (Table 5). These findings on modern methods of contraceptive use suggest that the above findings on the divisional variations of contraceptive use in rural areas of Bangladesh are very robust.

**Table 5:** Logistics regression estimates of contraceptive use (modern methods only) among women aged 15–49 in Bangladesh, 2014 BDHS^¥¥^.

Variables	Full Sample Odds Ratio [95% CI]	Urban Odds Ratio [95% CI]	Rural Odds Ratio [95% CI]
*Division*			
Chittagong	0.75** [0.64–0.88]	1.01 [0.70–1.46]	0.66** [0.55–0.78]
Dhaka	0.97 [0.82–1.15]	0.91 [0.64–1.30]	0.99 [0.82–1.21]
Khulna	1.09 [0.93–1.28]	0.98 [0.68–1.41]	1.13 [0.95–1.35]
Rajshahi	1.25** [1.06–1.47]	1.06 [0.74–1.51]	1.29** [1.08–1.54]
Rangpur	1.34** [1.12–1.61]	1.12 [0.78–1.60]	1.38** [1.13–1.67]
Sylhet	0.62** [0.52–0.75]	0.87 [0.59–1.28]	0.58** [0.48–0.71]
Barisal	[REF]	[REF]	[REF]
*Intercept*	*1*.57** *[1*.*16–2*.*11]*	*1*.97* *[1*.*13–3*.*42]*	*1*.65** *[1*.*15–2*.*37]*
*Total (N)*	*14292*	*3963*	*10329*

*p<0.05,

**p<0.01, [REF] indicates reference category;

^¥¥^ The odds ratios are adjusted for respondents’ age, religion, education, employment status, wealth index, access to media, number of visits by family planning workers, attitude towards ideal number of children, women’s autonomy and husband’s education; Data Source: The 2014 Bangladesh Demographic and Health Survey

## Discussion

The objectives of this study were to examine regional variations in contraceptive use in Bangladesh and to assess the extent to which the variations differ by place of residence (i.e., urban or rural). We hypothesized that there would be substantial variations in contraceptive use by divisions, and we also expected to find significant differences in the divisional variations in contraceptive use both in rural and urban areas. Consistent with our expectation, based on a full sample (i.e., rural and urban combined), it was found that there were significant differences in contraceptive use across divisions in Bangladesh. However, in contrast to our expectation, we did not find any significant difference in contraceptive use by division in urban areas of Bangladesh. But significant variations in contraceptive use across divisions were found in rural areas of Bangladesh. The significant variations in contraceptive use across rural areas may be due to differences in the prevalence of poverty (as shown in Table 1) [47], unequal allocation of resources and family planning activities across divisions in Bangladesh [11, 16, 18]. Thus, findings of the current study suggest that pragmatic approaches to family planning programs are needed based on local contexts and needs in order to increase the contraceptive use rates in the divisions that are lagging behind—in this case Chittagong and Sylhet.

What policy interventions should be taken to address the regional disparity in contraceptive use in rural areas of Bangladesh? First and foremost, emphasis should be given in allocating more resources [48] to rural areas compared to urban areas in general, and for rural areas of Chittagong and Sylhet divisions in particular. Then comes the question of which sectors should receive higher priority in maximizing the utilization of the allocated resources for increasing contraceptive use. Findings in the context of rural areas—that is, no significant difference in contraceptive use based on the variables of women’s education and employment status, and the inverse association between wealth and contraceptive use—suggest that interventions in other areas such as creating awareness among men and women about the negative consequences of having more children (e.g., on maternal and child health) and the benefits of having smaller families might be more effective in eliminating regional disparities in contraceptive use in rural areas of Bangladesh. This is supported by the fact that women in rural areas who mentioned three or more children as the ideal number of children to have were less likely to use contraceptives than those who considered having two or less than two children as the ideal number of children to have (Table 3). Thus, building awareness of the benefits of having a smaller family would exert a strong positive impact on increasing contraceptive use in rural areas of Bangladesh.

Findings of this study also suggest that another effective intervention for increasing the CPR is to increase family planning worker’s visits in rural areas focusing on the Chittagong and Sylhet divisions. We found a rate of contraceptive use that was 2.14 times higher among women in rural areas who had a family planning workers’ visit in the last six months in rural areas compared to those who did not have a visit (Table 4). Previous research revealed that a considerable number of current contraceptive users in Bangladesh had an incorrect perception about the negative impact of contraceptives such as their use posing serious damage to the user’s health, impairing fertility, and being unsafe for prolonged use [49]. Hence, expanding family planning workers’ visits to rural areas might have a positive impact on eliminating the incorrect perceptions about the negative effects of contraceptive use. This will eventually pave the way for increasing the CPR in rural areas of Bangladesh. In the 4th Health Sector Program (2017–2021), the Government of Bangladesh has given utmost priority in reducing regional disparities in contraceptive use by introducing regional family planning packages, particularly for the Sylhet and Chittagong divisions [50]. While this is a very good initiative, it is now more important to ensure delivering quality counselling and services through proper monitoring and evaluation in order to achieve the desired goal of reducing the regional disparity in contraceptive use.

Another effective indirect strategy to substantially reduce the regional disparities in contraceptive use in rural areas could be that of facilitating women’s autonomy. This is supported by the findings of this study, which are that in rural areas women with high level of autonomy were found to have 15.0% higher odds of contraceptive use compared to their peers with a low autonomy. This finding is also consistent with earlier research [14, 51–53]. In general, findings of these studies suggest that women’s autonomy essentially facilitates their greater role in decision making related to contraceptive use, and makes women more capable to overcome barriers related to contraceptive use such as resisting family and social pressure of having more children, avoiding misconceptions about contraception, and taking initiatives to deal with any side effects of using contraception [34, 54]. Studies have shown that participation in income generating activities, getting employed [55], partners’ positive attitude and participation in microfinance [56], among other factors, promote women’s autonomy in Bangladesh. Another aspect that should be taken into consideration is changing the mindset of the society—that is, the need for greater awareness among men as well in facilitating gender equality by their recognizing women’s role in the decision-making process. This is particularly important for patriarchal societies like Bangladesh where stereotyped attitudes towards women is one of the major obstacles for women’s autonomy [57, 58].

Moreover, the prevalence of child marriage among girls (i.e., married before the age of 18) in Bangladesh is another hindrance for enhancing women’s autonomy because child marriage has a wider negative impact on their education, their health, labour force participation and financial solvency [59–60]. Early childbearing and consequently having a higher number of children are also the outcomes of child marriage [61]. This is supported by the fact that the prevalence of adolescent motherhood is very high in Bangladesh with very limited to no decline in the rate of adolescent motherhood over time (33.0% in 1993–94 to 30.8% in 2014) [3]. One viable strategy to substantially reduce the prevalence of adolescent motherhood could be to increase the rate of contraceptive use substantially among adolescents in order to postpone their childbearing, which will minimize the above-mentioned negative consequences to a large extent. However, findings of this study showed a 31.0% of lower odds of using contraception among women ages 20 years and under in rural areas compared to their peers ages 20 years and over. Therefore, the family planning programs in Bangladesh should place more emphasize on providing family planning services to women ages 20 years and under in order to increase the rate of contraceptive use.

## Conclusion

The family planning programs in Bangladesh—which was once considered the role model for developing countries in particular—is facing daunting challenges in achieving the CPR target of 75.0% by 2021 in order to achieve the below replacement level of fertility. The challenges are even more acute because in some regions of Bangladesh the CPR has been much lower for several decades despite continuous interventions by the Government and Non-Government Organizations (NGOs). Findings of this study suggest that—even after adjusting for the selected sociodemographic and cultural characteristics of the respondents—Sylhet and Chittagong have lower rates of contraceptive use than Barisal, whereas Rajshahi and Khulna have higher rates of contraceptive use than the reference category. What other potential unobserved factors could provide plausible explanations for this paradox? Kibria and colleagues (18) identified factors that could contribute for scaling-up contraceptive use in Sylhet—the division with the lowest contraceptive use in Bangladesh. They revealed that the number of alive children, presence of a male child, husband’s higher education, receiving a visit from a family planning worker, and membership in a non-governmental organization would exert strong influences in increasing the rate of contraceptive use. While their study provides some direction for effective intervention, it would be worth investing in conducting qualitative research in divisions that have very high rates (in this case Rajshahi and Khulna) and very low rates of contraceptive use (in this case Chittagong and Sylhet). Such an in-depth study in the context of contrasting situations would facilitate uncovering unobserved factors behind the high rates of contraceptive use in some divisions and identifying the unobserved barriers of contraceptive use in other divisions that have a lower rate of contraceptive use in Bangladesh.

Some limitations of this study should be mentioned, however. First, poverty has been found to be one of the pivotal determinants of contraceptive use [62]. It would have been better to include the average household income in the regression analysis. However, due to limitations of the data, we could not include this variable in the analysis. Instead, we have used a wealth index as a proxy indicator of poverty, which has been widely used in earlier research [11, 16]. Second, the ultimate goals of successful family planning programs are to (1) reduce unintended pregnancies, (2) prevent unsafe abortions, (3) reduce infant mortality, and (4) slower the population growth. To achieve these goals, equal attention should also be given to addressing the discontinuation of contraception and the unmet need for family planning. However, this study did not focus on the regional disparity in discontinuation of contraception and the unmet need for family planning. Having better insight about the extent and causes of regional disparities in the discontinuation of contraception and the unmet need for family planning would better enable program planners to achieve the desired success of their family planning interventions. Hence, this aspect should receive extensive attention in future research.

Future research should also focus on two other aspects in greater detail. First, it should include more predictors of women’s autonomy (e.g., education, employment, and income) to create a better composite scale for women empowerment and then predict its impact on contraceptive use in Bangladesh. Second, it should carry out a separate analysis for rural and urban areas of each division by combining multiple surveys of the Bangladesh Demographic and Health Surveys. This will provide better insights for program planners and policy makers for each division. Despite these limitations, this study provides new insights about the extent of divisional variations of contraceptive use in rural and urban areas of Bangladesh. Findings of this study would provide directions and facilitate the adaption of a pragmatic approach for increasing contraceptive use in the divisions that are lagging behind.
